# Kawasaki Disease in Canada: The Impact of the COVID-19 Pandemic

**DOI:** 10.1016/j.cjcpc.2025.08.001

**Published:** 2025-08-06

**Authors:** Shannon Oliver, Michael Khoury

**Affiliations:** Department of Pediatrics, the University of Alberta, Edmonton, Alberta, Canada

**Keywords:** Kawasaki disease, COVID-19, epidemiology

Kawasaki disease (KD) remains the most common cause of pediatric acquired heart disease in high-resource countries.^1^ When left untreated, 25% of children with KD develop coronary artery aneurysms. However, if treated in a timely manner with intravenous immunoglobulin, the prevalence of coronary artery lesions decreases to approximately 4%.^1^ Since its initial description, there have been significant ongoing efforts to determine the underlying causes of KD. A leading suspected risk factor, among others, has remained a prodromal viral illness that is thought to trigger KD through adverse immune-mediated responses in genetically predisposed children.^2^ Thus, in early 2020, with the beginning of the COVID-19 pandemic, a unique impact on the prevalence of KD unfolded. First, with the introduction of social distancing, masking, and other precautions, the prevalence of non–SARS-CoV-2 viral infections decreased.^3^ Second, a distinct KD-like postinfectious immune-mediated response to COVID-19 was identified. For example, between February 18 and April 20, 2020, in the Bergano province of Italy, one of the epicenters for the COVID-19 pandemic, a 30-fold increase in patients presenting with a KD-like picture (and being managed as KD) was noted, with patients showing evidence of having had an immune response to the SARS-CoV-2 virus.^4^ This KD-like illness was subsequently defined as a separate entity, termed multisystem inflammatory syndrome in children (MIS-C).^5^

Internationally, retrospective evaluations of the incidence of KD throughout the pandemic showed a decrease in cases,^6^, ^7^, ^8^ with a study from Kobe, Japan, specifically noting a markedly reduced incidence in the last quarter of 2020 when compared with previous years, coinciding with decreases in non–SARS-CoV-2 viral infections.^7^ Furthermore, a study from the same region of Japan demonstrated a rise in KD from 2023 onward, corresponding with an increase in circulating viruses.^9^ The changing patterns in the prevalence of KD throughout the COVID-19 pandemic mirrors the reverse pattern in MIS-C diagnoses, which peaked in 2021 in the United States with 6.79 cases per million persons, with a subsequent decrease to 0.11 cases per million persons in 2023.^10^ Despite this international work, the impact of the COVID-19 pandemic on the prevalence of KD across Canada has not been well described to date.

In this issue of the *Canadian Journal of Cardiology, Pediatric and Congenital Heart Disease (CJCPC)*, Butris et al.^11^ evaluated the impact of the COVID-19 pandemic on the epidemiology of KD in Canada. To do so, they used administrative data from the Canadian Institute of Health Information between 2004 and 2023 and clinical data from patients admitted with acute KD at the Hospital for Sick Children in Toronto (estimated to comprise approximately 40% of KD cases nationally). They estimated the annual incidence of KD using provincial and national population estimates from Statistics Canada. A more granular evaluation of changes in demographic and clinical patient characteristics before, during, and after the COVID-19 pandemic was performed using the data from the Hospital for Sick Children.

There were 9228 acute admissions for KD over the 20-year study period. Although there were remarkably stable trends in KD incidence before the pandemic, the study identified a 30% reduction in the incidence of KD during the pandemic along with a disruption in seasonal variability, eventually returning to prepandemic patterns in more recent years.^11^ They report a change in presenting symptoms during the pandemic, compared with prepandemic KD cases, as well as an increase in incomplete KD toward the end of the pandemic. One of the key differences they note with regard to presenting symptoms was an increase in the number of patients presenting with abdominal pain in both the early and late pandemic period compared with prepandemic. This finding is of particular interest, considering that gastrointestinal involvement is a prominent feature of MIS-C.^12^

This paper builds on the published literature, providing the first Canadian experience of changes in KD patterns during the pandemic. Although their findings are consistent with studies from the United States, Japan, and South Korea,^6^, ^7^, ^8^ it is at odds with what was reported in Australia, where the incidence of KD remained stable in 2020 compared with the year prior.^13^ In 2020, Australia had a relatively low incidence of SARS-CoV-2 infections when compared with North American and European countries, with corresponding low diagnoses of MIS-C.^14^ As there is no specific test to diagnose MIS-C, in an effort to optimize sensitivity of diagnosis, the classification for MIS-C is quite broad, covering a wide variety of phenotypic presentations, including significant overlap with KD.^5^ It is therefore possible that, in countries experiencing higher incidences of SARS-CoV-2 exposure (and corresponding MIS-C illnesses), a number of true KD cases were misclassified as MIS-C, a finding potentially supported by a study by Bodnarchuk-Sokhatska et al.^15^ from Ukraine, which demonstrated that 46% of their MIS-C cases also met diagnostic criteria for KD. An additional explanation is that although Australia maintained strict border controls to keep the viral levels low, they also had the most number of days during the pandemic with no restrictions compared with other countries.^14^ Given the prevailing theory that the reduction in KD cases during the COVID-19 pandemic was due to public health measures such as masking and social distancing that resulted in a decrease in non–SARS-CoV-2 virus exposure in susceptible individuals, having more time with no restrictions and a “business as usual” approach may have meant a more normal viral exposure (and thus subsequent KD) pattern. The blunting of seasonal variability observed in the present study by Butris et al. supports the notion that the decreased prevalence of diagnosed KD cases in Canada is directly linked to a distinct period of reduced viral exposures.

An interesting finding reported by Butris et al. was that patients toward the end of the pandemic were more likely to present with incomplete KD.^11^ Although this may represent a true shift in pattern of clinical presentation, it may also represent misdiagnosis of MIS-C. The 2 most prevalent strains toward the latter part of the COVID-19 pandemic were Omicron and Delta, which were associated with a milder, more KD-like picture of MIS-C, when compared with the original strain.^16^ It is therefore possible that an increase in incomplete KD cases actually represented cases of MIS-C. Importantly, Butris et al. did not specifically evaluate the presence of a recent COVID-19 infection in their analysis. It is therefore difficult to make inferences about the possibility of MIS-C diagnosis among the identified KD cases. Specifically, it would have been interesting to evaluate the proportion of incomplete KD cases (compared with complete cases) that had an antecedent COVID-19 exposure. This represents an area for further study moving forward.

To conclude, this important work by Butris et al. in the current issue of *CJCPC* emphasizes 2 important points regarding the epidemiology of KD. The first is that the incidence of KD cases remains closely linked to the prevalence of viral exposures; the decreased incidence of non–SARS-COV-2 viruses early in the COVID-19 pandemic is likely responsible in large part to the decreased incidence of KD observed throughout much of the world (including Canada) during this time period. The second point is that, in the absence of diagnostic tests, diseases with overlapping phenotypes, such as KD and MIS-C, can potentially be interchangeably mistaken for each other and can confuse prevalence estimates. This poses unique challenges for surveillance programs and registries such as the International Kawasaki Disease Registry. These challenges also mirror the same diagnostic uncertainty we experience as clinicians when attempting to differentiate and provide care for patients presenting with postinfectious inflammatory syndromes, and highlight the ongoing need for further efforts to truly understand the biological basis of these entities.
